# A time-course transcriptome analysis of wax gourd fruit development reveals predominant genes regulating taste and nutrition

**DOI:** 10.3389/fpls.2022.971274

**Published:** 2022-09-08

**Authors:** Shudan Xue, Xiaotong Wan, Sen Lu, Yujuan Zhong, Dasen Xie

**Affiliations:** ^1^Guangdong Key Laboratory for New Technology Research of Vegetables, Vegetable Research Institute, Guangdong Academy of Agricultural Sciences, Guangzhou, China; ^2^Guangdong Laboratory for Lingnan Modern Agriculture, Guangzhou, China

**Keywords:** wax gourd, taste, nutrition, monosaccharides (glucose and fructose), malic acid, citrulline, transcriptome

## Abstract

Wax gourd, which belongs to Cucurbitaceae, is an excellent plant resource with the concomitant function of both medicine and foodstuff. Its unique taste and rich nutrition are deeply accepted by consumers. However, the main flavor and nutrients are still unclear, which restricts the quality breeding process of wax gourd. Here, we discovered that monosaccharides, malic acid and citrulline affect the flavor and nutrition of wax gourd and clarified the dynamic accumulation process of these metabolites. To gain insights into the underlying predominant genes regulating accumulation of these metabolites, we performed a time-course transcriptome analysis using RNA-sequencing analysis and compared the expression of screened genes among twenty-four germplasms with different metabolites levels. In addition, the expression abundance among the homologous genes were also analyzed. Finally, a total of 8 genes related to sugar [*AGA2* (*Bhi03G001926*), *SUS* (*Bhi12G001032*)], malic acid [*MDH* (*Bhi12G001426, Bhi01G000427*), *PEPC* (*Bhi12G000721, Bhi09G002867*), *ME* (*Bhi01G002616*)] and citrulline [*ASS* (*Bhi02G000401*)], respectively were determined. In summary, understanding the core genes influencing taste or nutrition will provide a theoretical basis for fruit quality improvement in wax gourd.

## Introduction

Wax gourd [*Benincasa hispida* (Thunb.) Cogn.], known as ash gourd or winter melon, is one of the most valuable plants in Cucurbitaceae family. Fruits of wax gourd are valued for nutritional and medicinal purposes, which are rich in water and valuable phytochemicals (sugars, organic acids, amino acid, vitamins, etc.) (Palamthodi and Lele, 2014; Sun et al., 2018). Various studies indicated that wax gourd had numerous therapeutic activities, including antihypertensive, antioxidant, anti-obesity, antiviral, anti-inflammatory and antitumor activities (Huang et al., 2004; Kumar and Vimalavathini, 2004; Nakashima et al., 2011; Doshi et al., 2015).

Wax gourd fruit stores a large amount of biomass in a very short growth cycle, along with changes in carbohydrate biosynthesis and metabolism (sugars and organic acid). Sugars and organic acids also majorly directly affect the organoleptic fruit quality and consumer acceptability. Soluble sugars, which mainly composed of disaccharide sucrose and its two hydrolysis products, the hexoses, such as glucose and fructose, are the source of sweetness in the fruits of Cucurbitaceae, and different sugar components result in different sweetness (Pangborn, 1963; Yamaguchi et al., 1970; Doty, 1976). The different sweetness of Cucurbitaceae family members mainly depends on the type and composition of sugar. Among them, watermelon, melon, pumpkin all have more sweetness than cucumber owing to with more sucrose. (Corrigan et al., 2000; Hu et al., 2009; Dai et al., 2011; Zhong et al., 2017; Gao et al., 2018; Muhammad Jawad et al., 2020; Umer et al., 2020). For several Cucurbitaceae species, many sugar genes associated with unloading and metabolism during development have previous reported, which composed of raffinose synthase and stachyose synthase (Rennie and Turgeon, 2009), α-galactosidase (Liu et al., 2022), soluble/insoluble acid invertase and neutral invertase (Chrost and Schmitz, 1997), sucrose phosphate synthase (Li et al., 2018), sucrose synthase, UDP-glucose 4-epimerase, UDP-galactose/glucose pyrophosphorylase, hexokinase, fructokinase, phosphoglucomutase and phosphoglucoisomerase and so on (Dai et al., 2011; Umer et al., 2020). Although genes related to fruit sugar quality have been reported in some gourds, few attempts have been made to detect the key genes that influence wax gourd sugar phenotypic changes.

Organic acids as crucial factors influence organoleptic quality and consumer demand. The types and quantity of organic acids varies considerably among different Cucurbitaceae family members. Citric acid is the predominant organic acid found in melon, with levels of about only 0.2% fresh weight, followed by malic acid levels (Leach et al., 1989; Wang et al., 1996; Flores et al., 2001; Burger et al., 2003; Tang et al., 2010). It was recognized that malic acid and citric acid serve as main organic acids in the mature watermelon fruit (Gao et al., 2018; Aslam et al., 2020; Muhammad Jawad et al., 2020; Umer et al., 2020). Three organic acids (citric, malic, and fumaric) have been identified in the pumpkin fruit with varied content in particular species and cultivars (Nawirska-Olszanska et al., 2014). Acid homeostasis is the outcome of acid synthesis and metabolism. Organic acids are intermediates in the tricarboxylic acid (TCA) cycle, associated with series of enzymes. These enzymes consist of phosphoenolpyruvate carboxylase (PEPC), citrate synthase (CS), aconitase (ACO), isocitrate dehydrogenase (IDH), malate dehydrogenase (MDH), and malic enzyme (ME). However, there are no studies on the acid composition and associated key enzyme genes involved in wax gourd.

Citrulline is naturally present in many cucurbits. Its nutraceutical and therapeutic potentials have previous reported in many researches. Citrulline, as the main amino acid of wax gourd, has a significant effect on vasodilation, widely used to treat high blood pressure (Huang et al., 2004; Nakashima et al., 2011). The citrulline homeostasis is the composite outcome of its synthesis and metabolism, inseparable from arginine metabolism. Based on the current researches information and gene annotations, the genes involved in the citrulline biosynthesis include carbamoyl phosphate synthetase (), NO synthase (*NOS*), Ornithine transcarbamoylase (*OTC*), N- acetylglutamate synthase (*NAGS*), N-acetylglutamate kinase (*NAGK*), N-acetylglutamatyl-5-P reductase (*NAGPR*), N-acetylornithine aminotransferase (*NAOAT*), acetylornithine deacetylases (*NAOD*), while genes involved in citrulline catabolism consist of argininosuccinate synthases (*ASS*) and arginosuccinate lyase (*ASL*) (Winter et al., 2015; Joshi and Fernie, 2017). Other than watermelons, there is limited information available about the citrulline content and closely related genes in wax gourd.

Most wax gourd cultivars bear a giant fruit, which can weigh normally over 20 kg (Xie et al., 2019) or in some cases up to 50 kg (Dhillon et al., 2016). Thus, the main type of carbohydrate in wax gourd catches our attention and this study will focus on the types and proportion of sugar, acid and citrulline and the related genes. For sugar, organic acid and citrulline, many studies have previously reported about their biosynthesis, degradation and developmental regulation of the genes/enzymes in some Cucurbitaceae family members, but comprehensive metabolite profiling of sugars, organic acids and citrulline in wax gourd is lacking. Nowadays the high-quality wax gourd draft genome sequence and the available databases of genomic information [Cucurbit Genomics Database (CuGenDB)] would likely provide a genetic information foundation for exploring metabolism networks of flavor and nutrition metabolites (Xie et al., 2019). Therefore, we performed comparative analysis of primary metabolites and transcriptome changes during the fruit developmental progress under significantly different varieties, which would provide clear insights into the synthesis, catabolism of sugar, organic acid and citrulline in wax gourd. Furthermore, our study establishes a foundation for flavor and nutritional quality improvement in wax gourd breeding.

## Materials and methods

### Plant materials and fruit samples

Two varieties (B and G) with different quality phenotype (flavor and nutrition) were employed in this study, which were planted in the research experiment field of Vegetable Research Institute, Guangdong Academy of Agricultural Sciences. To explore the dynamic changes of wax gourd fruits sugar, organic acids and citrulline at time-course developmental stages, the flesh samples on the middle section of two varieties fruits were collected at 0, 5, 10, 20, 30 days after pollination (commercial maturity stage), respectively. Twenty-four germplasms were used to analyze the correlation between the expression of key genes and the content of metabolites. Three or six individual fruits from different plants were chosen at each time point or different germplasms and all the samples were divided into two subsets. One subset was freeze-dried to a powder for sugar, organic acid and citrulline content determinations. The other subset was immediately frozen in liquid nitrogen and stored at −80°C for transcriptome analysis or RT-PCR verification.

### Measurement of sugar, organic acid and citrulline content in wax gourd fruit flesh

The frozen fruit samples were lyophilized and used for the following preparation. Determination of soluble sugars was carried out according to Obando-Ulloa et al. (2009), with slight modifications (Obando-Ulloa et al., 2009). High-performance liquid chromatography (Alliance e2695 HPLC system, Waters, Milford, MA, United States) coupled with a refractive index detector (HPLC-RID) was applied to determine the sugar profile of samples. The lyophilized powder (20 mg) was extracted with 50% acetonitrile water and soluble sugar concentration was determined in a medium polarity NH_2_ column (Waters Xbridge BEH Amide-4.6 × 250 mm, 5 μm particle size), using acetonitrile/deionized water in a 80:20 v/v combination as the mobile phase with a flow of 1 ml/min. Glucose, fructose and sucrose purchased from Sigma-Aldrich (St. Louis, MO, United States) were used as calibration standards.

Organic acids, including malic acid, shikimic acid, citric acid and fumaric acid, were detected according to Scherer et al. (2012) with slight modifications (Scherer et al., 2012). High-performance liquid chromatography (Alliance e2695 HPLC system, Waters, Milford, MA, United States) coupled with Waters 2998 photodiode array detector (HPLC-PDA) was applied to determine organic acids. A sample of 20 mg lyophilized powder was extracted with distilled deionized water. Organic acids were determined in reverse-phase C18 column (Waters Atlantis T3 C18 column, 250 mm × 4.6 mm i.d., 5 μm particle size) operated at 25°C, using 5 g/l (NH4)_2_HPO4 solution (pH 2.5)/methanol in a 97:3 v/v combination as the mobile phase with a flow of 0.6 ml/min. Eluted compounds were detected by UV absorbance at 214 nm and quantitated by external linear calibration. Malic acid, shikimic acid, citric acid and fumaric acid purchased from Sigma-Aldrich (St. Louis, MO, United States) were used as calibration standards.

HPLC-PDA equipped with a C18 column (Phenomenex, Gemini C18, 250 × 4.6mm, 3 μm partial size) was carried out to detect citrulline (Jayaprakasha et al., 2011). A sample of 20 mg lyophilized powder was extracted with MeOH/1N HCl in a 30:1 v/v combination and the supernatants were filtered through a 0.22-μm membrane filter. The column temperature was set at 25°C and detection was performed at 207 nm, using 0.03mM phosphoric acid as the mobile phase with a flow rate of 0.7 ml/min. L-citrulline purchased from Sigma-Aldrich (St. Louis, MO, United States) were used as calibration standards.

### RNA-seq and read mapping

Total RNA was extracted from the frozen wax gourd flesh at 0, 5, 10, 20, 30 DAP from B variety and 30 DAP from G variety using a Huayueyang RNA extraction kit (Huayueyang, Beijing, China) according to the manufacturer’s instructions. At each time point, six fruit harvested from different plants growing in consistent conditions were divided into two biological replicates. The quantity, quality, and integrity of the twelve RNA samples were determined with an Agilent 2100 Bioanalyzer and a Nanodrop NanoPhotometer. RNA-seq was performed on an Illumina Nova Seq 6000 Platform at Novogene Corporation Inc., and low-quality reads and adapters were removed by the company. High-quality reads were mapped to the wax gourd B227 reference genome^1^ (Xie et al., 2019) using Hisat2 (v.2.0.5) (Kim et al., 2015) with default settings. Gene expression was estimated in terms of fragments per kilobase of transcript per million mapped reads (FPKM).

### RT-qPCR analysis

Total RNA was isolated using a Huayueyang RNA extraction kit (Huayueyang) and then reverse transcribed using SuperReal PreMix Plus (Tiangen, beijing) following the manufacturers’ protocol. RT-qPCR was conducted in 96-well plates with a CFX connect Real-Time PCR Detection System (Bio-Rad, United States) using the Ultra SYBR Mix kit (CWBIO, Beijing, China). Three biological replicates and three technical replicates were performed for each combination of cDNA samples and primer pairs. Relative quantitative analysis of data was performed by the 2^–ΔΔ*Ct*^ method with internal control gene β-actin. The gene-specific RT-qPCR primers are listed in Supplementary Table 9.

### Statistical analyses

The experimental data were analyzed using SPSS 23.0 (company, city, state abbrev if United States, country). For weighted gene co-expression network analysis (WGCNA), differentially expressed genes (DEGs) (coefficient of variation (CV) > 0.5) were used to generate co-expression network modules by WGCNA package in R. The co-expression modules were obtained using automatic network construction function (blockwiseModules) with default parameters, apart from the soft threshold power of 10, TOMtype was signed, mergeCutHeight was 0.25 and minModuleSize was 30. And the visio2021 and Mev 4.2 software were used for drawing pathway and heat map analysis of gene expression.

## Result

### Metabolism of sugar, acid and citrulline in wax gourd during fruit development

**FIGURE 1:**
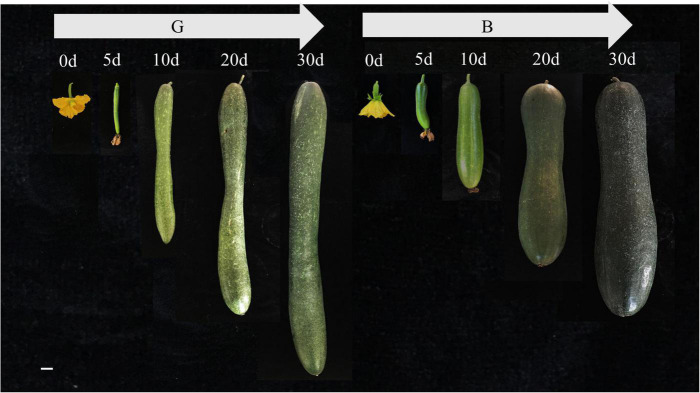
The developmental process of two specific significantly different varieties (G and B) at 0, 5, 10, 20, 30 days after pollination, respectively. Bar = 3 cm.

**FIGURE 2:**
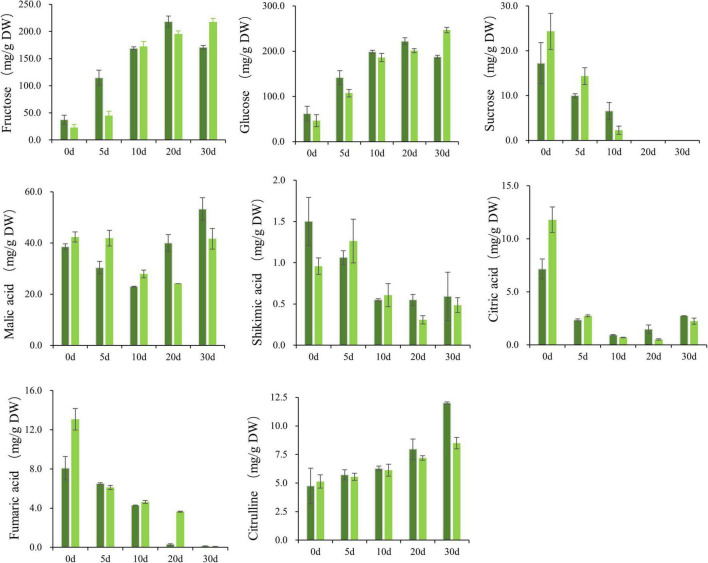
Soluble sugars (glucose, sucrose, and fructose), organic acids (malic acid, fumaric acid, citric acid, and shikimic acid) and citrulline in wax gourd fruit at 0, 5, 10, 20, and 30 days after pollination between two specific significantly different varieties. Each value indicates mean ± standard deviation (± SD) values of three biological replicates. The concentrations were expressed in milligram per gram dry weight (DW). Dark green and light green represent B, G varieties with diverse genetic backgrounds.

Sugar increases gradually in the developmental stage of wax gourd fruit (Figure 1). The main components of sugar in the young fruit stage are fructose, glucose and sucrose, but in the commodity maturity stage, fructose and glucose are the main components, with sucrose reduced to an undetectable level. As shown in the Figure 2, for B variety, with fruit enlargement and development, fructose and glucose reached the maximum at 20 days after pollination, and decreased slightly at 30 days after pollination. For G variety, hexoses (fructose, glucose) gradually accumulated and reached the peak at 30 days after pollination. The sugar content of the two varieties was significantly different at 30 DAP, showing that G was significantly higher than B.

The study found that the main organic acid in wax gourd fruit is malic acid, and its accumulation pattern is “V”-shaped, showing a trend of first decreasing from 0 to 10 DAP, and then increasing steadily as the fruit matured. For fumaric acid, citric acid, and shikimic acid, these acids are higher in the young fruit stage and lower in the commercial maturity stage. The malic acid content of the G variety at the later stage of development was significantly lower than that of the B variety (Figure 2).

As indicated in Figure 2, there was an increase of citrulline gradually during the course of fruit development. With fruit enlargement and development, a maximum of citrulline content was reached the final values of nearly 11.97 and 8.52 mg⋅g^–1^ dry weight at 30 DAP, respectively for B and G.

### The generation of fruit development time course transcriptome data

To gain insights into wax gourd fruit development, we generated time-coursed transcriptome data by sampling the middle section flesh at 0 DAP, 5 DAP, 10 DAP, 20 DAP, 30 DAP from a representative wax gourd variety (B). To further narrow down the key genes related to fruit flavor and nutrition, another variety (G) at the commercial maturity stage 30 DPA was also sampled.

A total of 559 million high-quality reads was generated and mapped to the wax gourd B227 reference genome using Hisat2 (v.2.0.5) (Kim et al., 2015; Xie et al., 2019). About 7 Gb of clean bases for each individual sample was obtained with more than a 94% Q30 base percentage. And the average GC content was 43.14% for all libraries. An average of 92% reads were uniquely mapped (Supplementary Table 1) and used to calculate normalized gene expression level as fragments per kilobase of transcript per million mapped reads (FPKM). A comparison of the biological replicates showed that the expression values were highly associated (Supplementary Figure 1), indicating a high repeatability of the collection process. Therefore, the average FPKM value was used for expression analysis. In addition, six genes related with sugar, organic acid, citrulline metabolic pathways were randomly selected for qRT-PCR. Most of the genes showed a consistent expression profile between qRT-PCR and RNA-seq (Supplementary Figure 2), which indicated that the transcriptome data were of high quality and reliable.

Based on the pairwise comparison with | log2(fold change)| ≥ 0 and FDR value < 0.05 as the threshold, a total of 937 (546 up- and 391 down-regulated), 6019 (3073 up- and 2946 down-regulated), 11691 (5664 up- and 6027 down-regulated), 10605 (5060 up- and 5545 down-regulated), 2519 (1301 up- and 1218 down-regulated), 10222 (4927 up- and 5295 down-regulated), 8269 (3852 up- and 4417 down-regulated), 7058 (3228 up- and 3830 down-regulated), 4544 (1753 up- and 2791 down-regulated), 7489 (3473 up- and 4016 down-regulated) and 4718 (2356 up- and 2362 down-regulated) differentially expressed genes (DEGs) were identified in B_5d vs. B_0d, B_10d vs. B_0d, B_20d vs. B_0d, B_30d vs. B_0d, B_10d vs. B_5d, B_20d vs. B_5d, B_30d vs. B_5d, B_20d vs. B_10d, B_30d vs. B_10d, B_30d vs. B_20d and G_30d vs. B_30d, respectively using DESeq version 2 (Supplementary Tables 2, 3).

GO enrichment analysis of wax gourd fruit developmental progress was conducted, and the result indicated the differentially expressed genes were mainly enriched in cellular carbohydrate metabolic/biosynthetic process, cellular glucan metabolic process, cellular polysaccharide metabolic process, cellulose metabolic/biosynthetic process, amide biosynthetic/metabolic process, peptide biosynthetic/metabolic process during fruit development, as shown in Supplementary Table 4 in detail.

### Candidate genes associated with sugar accumulation of wax gourd fruit

**FIGURE 3:**
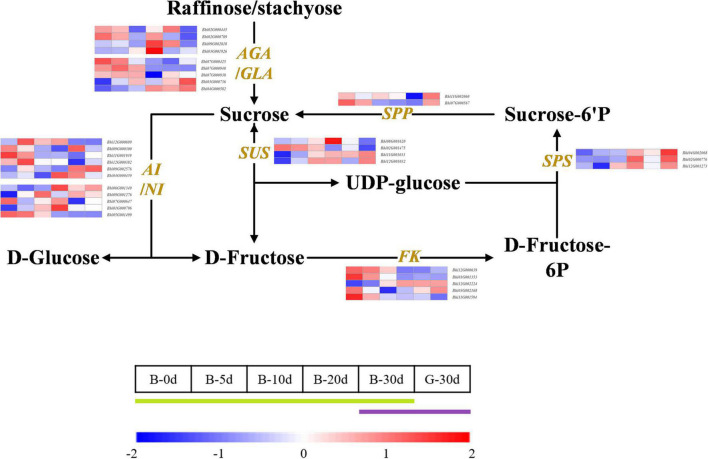
Developmental expression profiles of genes of sugar metabolism in developing wax gourd fruit. *AGA/GLA*: alkaline/acid α-galactosidase; *AI/NI:* acid/neutral invertase; SPP: sucrose-phosphatase; *SPS*: sucrose phosphate synthase; *SUS*: sucrose synthase; FK: fructokinase.

Wax gourd is rich in monosaccharides, which affect its flavor and taste. In order to explore the key genes involved in regulating sugar accumulation in wax gourd, we mapped the KEGG pathway of sugar anabolism in wax gourd (Figure 3). Based on transcriptome data and gene annotation, we searched for all the homologous genes of key enzymes in the pathway nodes (Supplementary Table 5). Based on the expression difference between the two varieties at the commercial stage of 30 DAP and the expression difference in the developmental period of one variety, we found that the enzymes that positively regulate the accumulation of fructose and glucose are α-galactosidase, invertase, sucrose synthase, but different homologous genes of the same enzyme may have specific functions, of these, α-galactosidase (*BhAGA2* (*Bhi03G001926*), *Bhi03G000736, Bhi04G000582*), invertase (*Bhi12G000699, Bhi06G001349, Bhi01G000796*) and sucrose synthase (*Bhi11G001031, Bhi12G001032*) positively regulate monosaccharide content. In addition, during fruit expansion and development, sucrose gradually decreased, and the expression of sucrose-phosphatase genes (*Bhi11G002060, Bhi07G000567*), which positively regulate sucrose accumulation, also decreased with development. Fructokinase is also an enzyme that negatively regulates monosaccharides, among which *Bhi11G001594* gradually decreases with the developmental stage, and its expression level in G variety is lower than that in B variety (Figure 3).

**FIGURE 4:**
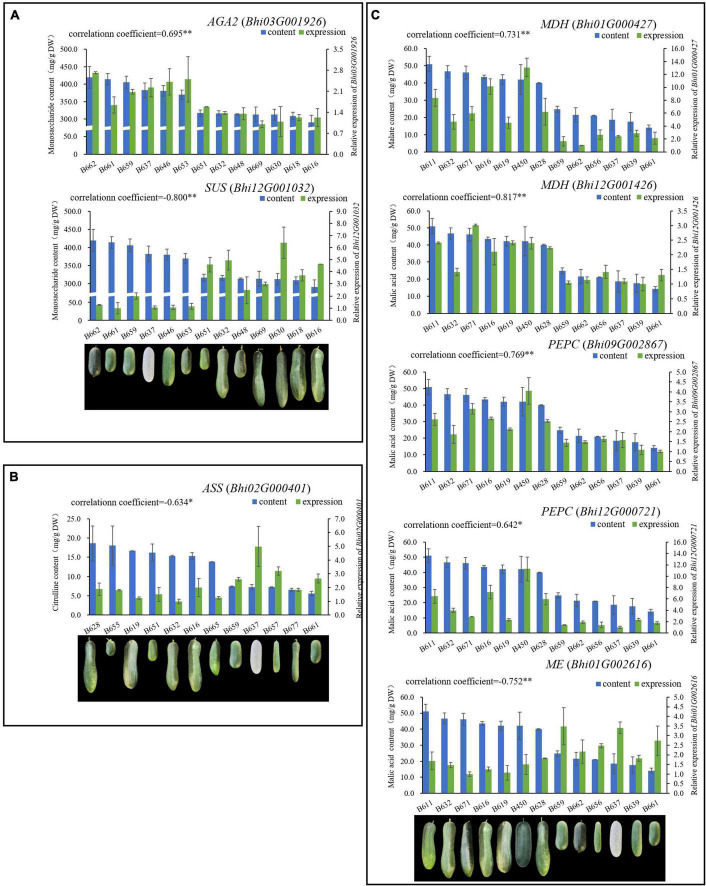
A total of 8 genes related to sugar [*AGA2* (*Bhi03G001926*), *SUS* (*Bhi12G001032*)], malic acid [*MDH* (*Bhi12G001426, Bhi01G000427*), *PEPC* (*Bhi12G000721, Bhi09G002867*), *ME* (*Bhi01G002616*)] and citrulline [*ASS* (*Bhi02M000401*)], respectively were determined, expression of which have high correlation with metabolite contents. **(A)** Genes related to sugar; **(B)** genes related to citrulline; **(C)** genes related to malic acid.

The taste of wax gourd and the composition and pattern of sugar accumulation are similar to those of cucumber. In the research of cucumber, the key enzymes regulating the accumulation of hexose are mainly α-galactosidase, sucrose synthase or invertase (Liu et al., 2022). Therefore, the expressions of the screened 8 genes associated with wax gourd monosaccharide from the above were compared among thirteen germplasms with different sugar levels. Finally, only *BhAGA2* (*Bhi03G001926*) expression was positively correlated with monosaccharide content, with a correlation coefficient of 0.695(*P* ≤ 0.01) (Figure 4A and Supplementary Figure 3). In addition, by comparing the expression abundance of these 8 genes in the homologous genes, it was also found that, *BhAGA2* (*Bhi03G001926*) has the highest expression abundance among the homologous genes and is a key gene regulating the accumulation of monosaccharides in wax gourd. Incredibly, one *SUS*-related gene (*Bhi12G001032*) is highly negatively correlated with monosaccharides among thirteen germplasm resources (*r* = −0.800, *P* ≤ 0.01) (Figure 4A).

### Candidate genes associated with organic acids accumulation of wax gourd fruit

**FIGURE 5:**
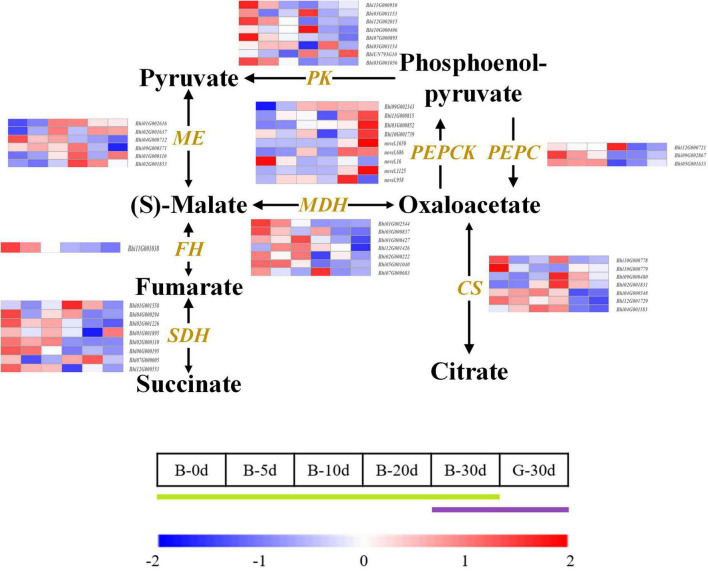
Developmental expression profiles of genes of organic acid biosynthesis and catabolism in developing wax gourd fruit. *PEPC*: phosphoenolpyruvate carboxylase; *PEPCK*: phosphoenolpyruvate carboxykinase; *MDH*: malate dehydrogenase; *ME*: malic enzyme; *PK*: pyruvate kinase; *SDH*: succinate dehydrogenase; *FH*: fumarate hydratase; *CS*: citrate synthase.

**FIGURE 6:**
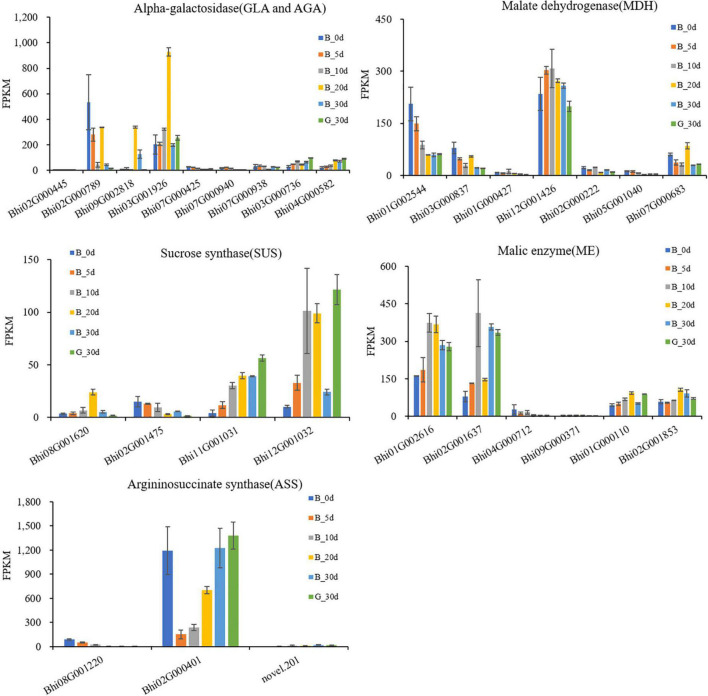
RNA-Seq expression profiles *of AGA/GLA, SUS, MDH, ME, ASS* family genes at different developmental stages in B fruit from 0 to 30 DAP, G fruit at 30 DAP.

The main type of organic acid in wax gourd fruit is malic acid, which shows a V-shaped accumulation trend during fruit growth and development. In order to explore the key genes involved in regulating the accumulation of organic acids in wax gourd, we also draw the organic acid KEGG pathway and compare the pathway associated genes (Figure 5 and Supplementary Table 6). The study showed that malic enzyme (ME) and malate dehydrogenase (MDH) are the key enzymes involved in the regulation of malic acid, among which ME is the enzyme responsible for cleaving malic acid. There are 6 *ME* homologous genes with different expression trends. Among them, expressions of *Bhi01G002616* and *Bhi01G000110* present inverted V-shaped trend accompanied by negatively correlation with malic acid during fruit developmental stages, moreover *Bhi01G002616* was found with the highest expression abundance among the *ME* homologous genes, therefore it is speculated as the core *ME* gene to regulate malic acid accumulation (Figure 6). Malate dehydrogenase (MDH) is a pivotal enzyme that positively regulates the accumulation of malate. It has 7 homologous genes with different expression trends. According to the difference in expression between the two varieties in the commercial period of 30 days, it is found that *Bhi12G001426, Bhi01G000427* and *Bhi02G000222* are the regulators for promoting malic acid accumulation. Among them, the expression of *Bhi12G001426* is highly expressed in *MDH* homologous genes (Figure 6). The homeostasis of malic acid is regulated by both biosynthesis and catabolism. Phosphoenolpyruvate carboxylase (PEPC) catalyzes the direct conversion of phosphoenolpyruvate (PEP) to oxaloacetate (OAA), thereby reducing the carbon flux to pyruvate and other pathways, and is an upstream core gene that regulates the accumulation of organic acids. *PEPC* has 3 homologous genes (*Bhi12G000721, Bhi09G002867, Bhi05G001633)*, mainly participate in organic acids metabolism (Figure 5).

The accumulation of citric acid in wax gourd showed a decreasing trend during the time course development, and the young fruit stage was significantly higher than the commercial fruit stage. Citrate synthase (CS) was the key enzyme affecting the accumulation of citric acid. Among them, the expression of *Bhi10G000779* was positively correlated with the citric acid content during development (Figure 5).

Fumaric acid showed a decreasing trend during development, among which nine succinate dehydrogenase gene orthologs (SDH) were found to be differentially expressed, of these, the expression of *Bhi02G001226,Bhi02G000310,Bhi06G000195* was positively correlated with the accumulation of fumaric acid. In addition, by comparing the trends of those gene expression and fumaric acid content between 30 PDA of two materials (B and G), it was found that only *Bhi02G001226* and *Bhi06G000195* were the key genes positively regulating fumaric acid (Figure 5).

Malic acid is the predominant acid component that affects the taste of wax gourd, so the above eight screened genes associated with malic acid, *ME* (*Bhi01G002616, Bhi01G000110*), MDH (*Bhi12G001426, Bhi01G000427, Bhi02G000222*) and *PEPC* (*Bhi12G000721, Bhi09G002867, Bhi05G001633*), were compared about their expression among thirteen germplasm resources with different malic acid content (ranging from 14.16 mg/g dry weight to 50.97 mg/g dry weight, showing in Figure 4). Finally we found that the expression of *MDH* (*Bhi12G001426, Bhi01G000427*) and *PEPC* (*Bhi12G000721, Bhi09G002867*) were positively correlated with malic acid content (correlation coefficient, *r* = 0.817, 0.731, 0.642, 0.769, *P* ≤ 0.01, ≤0.01, ≤0.05, ≤0.01, respectively), the expression of *ME* (*Bhi01G002616*) is negatively correlated with malic acid content (correlation coefficient, *r* = −0.752, *P* ≤ 0.01). These five genes are the most decisive genes regulating the accumulation of malic acid in wax gourd (Figure 4C and Supplementary Figure 4).

### Candidate genes associated with citrulline accumulation of wax gourd fruit

**FIGURE 7:**
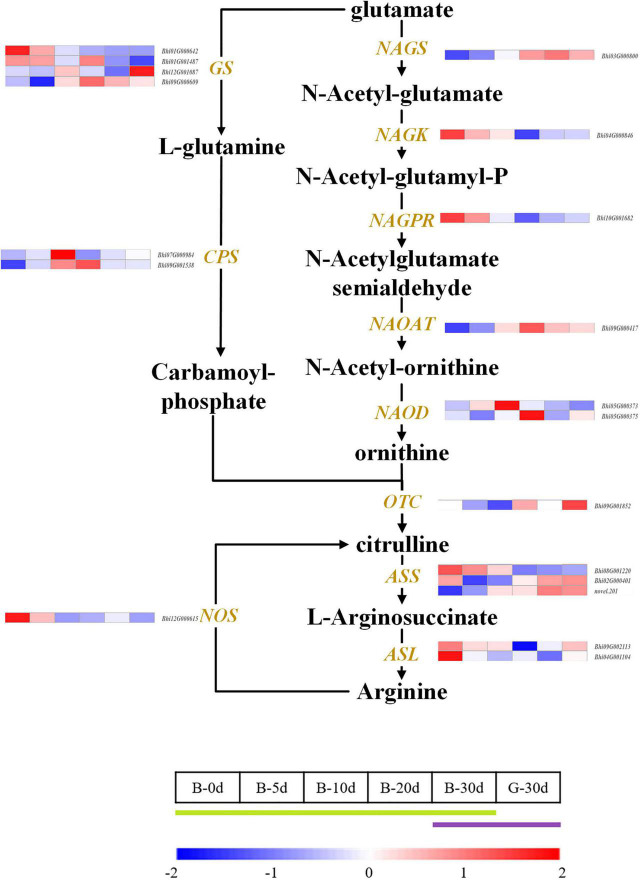
Developmental expression profiles of genes of citrulline biosynthesis and catabolism in developing wax gourd fruit. *NAGS*: *N*-acetylglutamate synthase; *NAGK: N*-acetylglutamate kinase; *NAGPR*: *N*-acetylglutamatyl-5-P reductase; *N*AOAT: *N*-acetylornithine aminotransferase; *NAOD*: *N*-acetylornithine deacetylase; *OTC*: ornithine transcarbamylase; *ASS*: argininosuccinate synthase; *ASL*: argininosuccinate lyase; *GS*: glutamine synthetase; *CPS*: carbamoyl phosphate synthetase; *NOS*; putative nitric-oxide synthase.

Citrulline showed an increasing trend during fruit development. The pathway was drawn based on the clear citrulline metabolism, and putative all homologous genes likely to be involved in the citrulline metabolic pathway were identified based on the wax gourd genomic and transcriptomic database (Figure 7 and Supplementary Table 7). Based on the expression difference between the two varieties at the commercial stage of 30 days and the expression difference in the developmental period of one variety, we found that the enzyme genes that positively regulate the accumulation of citrulline are N-acetylglutamate synthase (*NAGS*: *Bhi03G000800*), N-acetylornithine aminotransferase (*N-AOAT*: *Bhi09G000417*), and carbamoyl phosphate synthetase (*CPS: Bhi09G001538*) (Figure 7). But gene expression was significantly uncorrelated with citrulline content among natural germplasms with different citrulline (ranging from 5.6 mg/g dry weight to 18.63 mg/g dry weight) (Supplementary Figure 5). The breakdown of citrulline in plants is triggered by two catabolic enzymes, argininosuccinate synthases (ASS) and argininosuccinate lyases (ASL) (Figure 7). Of these, only the expression of one homologous gene *ASS* (*Bhi02M000401*) were negatively correlated with citrulline content among germplasm resources with different citrulline content (correlation coefficient, *r* = −0.634, *P* ≤ 0.05) (Figure 4B and Supplementary Figure 5). In addition, *ASS* (*Bhi02M000401*) had the highest expression abundance, whereas the other two *ASS*-related genes remained at low levels during all development stages (Figure 6).

### Co-expression network construction

**FIGURE 8:**
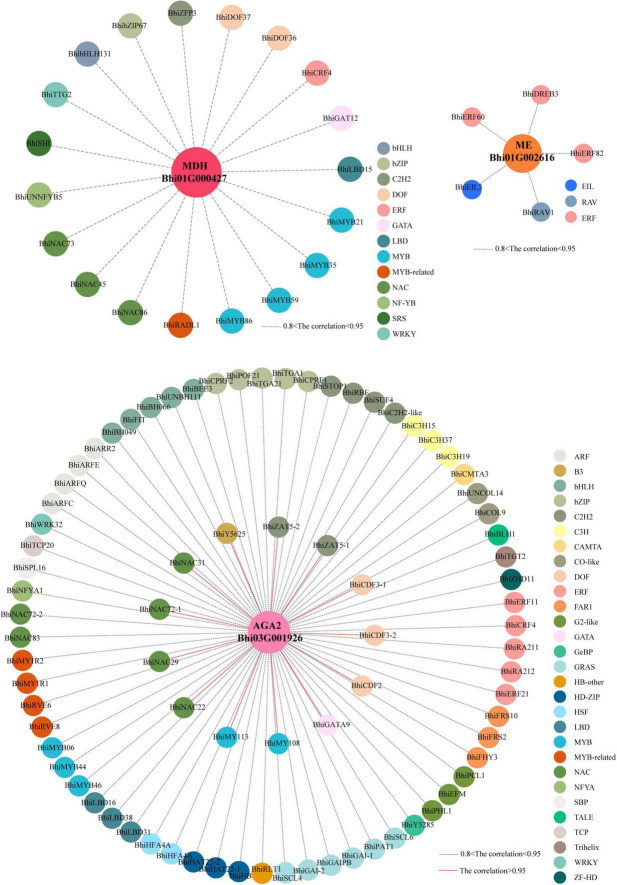
Coexpression networks of TFs and enzyme genes involved in taste and nutrition metabolites. Center circles represent key enzyme genes involved in soluble sugars (pink) and malic acid (dark-pink and orange) metabolism during fruit development. Outer circles with different colors represents different families of transcription factors identified in the same module whose transcripts are correlated with expression of enzyme genes. Red lines represent correlations higher than 0.95; dotted blank lines represents correlation between 0.8 and 0.95.

To further reveal the gene regulatory network of taste and nutrition metabolites in wax gourd fruits, weighted gene co-expression network analysis (WGCNA) was performed on the transcriptome data to investigate highly coordinated gene sets during fruit development. A total of 21 co-expression modules (each labeled with a different color) were identified based on their similar expression patterns (Supplementary Figure 6). In the above study, the key enzyme genes screened based on metabolic pathways belong to different modules, namely, sugar gene *AGA2* (*Bhi03G001926*) belongs to the “yellow” module, malic acid genes *MDH* (*Bhi12G001426, Bhi01G000427*) belongs to the “darkred and brown” module, malic acid gene *ME* (*Bhi01G002616*) belongs to the “purple” module, and citrulline gene *ASS* (*Bhi02G000401*) belongs to the “black” module. To further characterize the transcription factors (TFs) that putatively regulate taste and nutrition metabolites, we screened out 80 TFs with highly correlated with sugar gene *AGA2* (*Bhi03G001926*) in the yellow module and formed a correlation network (*R* > 0.80), among which, genes with correlation coefficients greater than 0.95 include 14 TFs (2 C2H2s, 3 Dofs, 4 NACs, 1 ZF-HD, 2 MYBs, 1 GATA, 1 B3). And we identified 19 TFs (3 NACs, 4 MYBs, 1 ERF, 1 GATA, 2 Dofs, 1 SRS, 1 bHLH, 1 WRKY, 1 C2H2,1 LBD, 1 NF-YB, 1 bZIP) highly correlated with malic acid genes *MDH* (*Bhi01G000427*) in the “brown” module and 5 TFs (2 ERFs, 1 EIL, 1 RAV) highly correlated with malic acid gene *ME* (*Bhi01G002616*) in the “purple” module(*R* > 0.80), but there is no transcription factor with a correlation coefficient greater than 0.8 with the ASS gene in the “black” module (Figure 8 and Supplementary Table 8). To sum up, these TFs, as highly connected genes, may have regulatory effects on accumulation of the taste and nutrition metabolites in wax gourd fruit development.

## Discussion

Wax gourd, which belongs to Cucurbitaceae, is an excellent plant resource that has the concomitant function of both medicine and foodstuff. Elucidation of the key genes influencing the fruit quality of wax gourd becomes essential because of higher consumer demand for quality. Sensory quality is mainly determined by fruit sugar and organic acid levels, in addition to the volatile aromatic components. The sweetness of wax gourd depends on fructose and glucose, and the acidity depends on malic acid. These three substances generally affect the flavor and taste of wax gourd. Citrulline, as the main amino acid of wax gourd, was widely used in traditional Chinese medicine to treat high blood pressure related to the NO pathway. (Huang et al., 2004; Nakashima et al., 2011).

### *AGA2* as the key gene regulating sugar accumulation in wax gourd

In wax gourd, the total sugar accumulated gradually with the developmental period, which was consistent with the sugar accumulation pattern of cucumber, watermelon and other cucurbit crops. Cucurbitaceae fruit sugar is first assimilated by the source organ, transported to the sink organ through the phloem, and finally accumulated in the fruit (Chrost and Schmitz, 1997),and raffinose and stachyose are the principal sugars for translocation from the source to the sink in Cucurbitaceae rather than sucrose (Zhang et al., 2012). Alpha-galactosidase is the main enzyme hydrolyzing stachyose and raffinose and determining sink strength in cucurbit plants (Gao and Schaffer, 1999). In Cucurbitaceae two species of a-galactosidase have been identified: acid α-galactosidase activity with acid pH optimum and alkaline α-galactosidase activity with a distinct alkaline pH optimum (Pharr and Sox, 1984). As the initial step in catabolism of the raffinose oligosaccharides, the distribution and function of these α-galactosidases seem to be different. In this study, based on the expression differences of transcriptome data, the expression trends of all α-galactosidase family genes in the wax gourd genome were clarified, including four alkaline α-galactosidases and six acid α-galactosidases. Among them, the expression abundance of *BhAGA2* (*Bhi03G001926*) gene is extremely higher than that of the other 9 homologous genes. The expression is highly correlated with sugar content in developmental stages and in differentiated germplasm. It is speculated to be a key factor regulating the sugar accumulation in wax gourd fruit. Alkaline α-galactosidase which contributes to sugar accumulation in fruit flesh has been reported in some Cucurbitaceae, such as cucumber (*Cucumis sativus* L.) and watermelon (*Citrullus lanatus* L.) and muskmelon (*Cucumis melo* L.). In watermelon, based on a genome-wide association study (GWAS) and functional analysis, the alkaline α-galactosidase *ClAGA2* functions in the hydrolysis of raffinose family oligosaccharides (RFOs) and regulates the accumulation of fruit sugar (Ren et al., 2021). In cucumber, *CsAGA2* (alkaline α-galactosidase 2) was involved in phloem unloading (Li et al., 2021), meanwhile overexpression of *CsAGA2* could alter the sugar metabolism resulting in substantial accumulation of monosaccharides, such as glucose and fructose (Liu et al., 2022). In melon, predominantly *CmAGA*2, Homologous gene of wax gourd *BhAGA2*, account for all of a-gal gene expression in the fruit tissue functioning in key processes of galactosyl-oligosaccharide metabolism (Carmi et al., 2003; Dai et al., 2011).

In contrast, the monosaccharides presented a noticeable positive correlation with sucrose synthase (*SUS: Bhi12G001032*) expression during the fruit time-course development, but showed an obviously negative correlation among natural germplasms at commercially available ripe stage with different sugar content Explanations for this may be that the different regulation mechanism between young and mature fruit stage, which needs further study.

In contrast, the monosaccharides presented a noticeable positive correlation with sucrose synthase (*SUS: Bhi12G001032*) expression during the fruit time-course development, but showed an obviously negative correlation among natural germplasms at commercially available ripe stage with different sugar content Explanations for this may be that the different regulation mechanism between young and mature fruit stage, which needs further study.

### Five genes related-*PEPC, MDH, ME* as the core genes regulating organic acid accumulation in wax gourd

Fruit acidity is due to the presence of organic acids, among which malic and citric acids are the main acids found in most ripe fruits (Etienne et al., 2013). As the main organic acid in watermelon, patterns of malic acid accumulation from 10 to 34 DAP in fruit development showed an inverted V shape, with decreasing trend in the commercial maturity stage (Gao et al., 2018; Aslam et al., 2020; Muhammad Jawad et al., 2020). Citric acid was the main organic acid in melon fruit, and its accumulation showed an increasing trend in the development process, but with slightly decreasing in the ripening period (Flores et al., 2001; Burger et al., 2003; Tang et al., 2010). Whether watermelon or melon, the research on organic acids in young fruit stages before 10 DAP is rarely reported. In our present study, the accumulation of organic acids was well monitored at successive developmental stages after ovary pollination (0, 5, 10, 20, 30 DAP). We found that wax gourd has a high malic acid content at 0 DAP, and then gradually decreased, reaching to minimum content around 10 ∼20 DAP, and then increased steadily as the fruit matured. With wax gourd fruit enlargement, the stage that biomass accumulates rapidly is around 10 ∼20 DAP. At this stage, the cell division of wax gourd is extremely fast, and cell skeleton structures such as cell walls are produced in large quantities. The cell wall is composed of a diversity of polysaccharides, such as cellulose, hemicellulose, or pectin (Caffall and Mohnen, 2009). So the carbon flux to polysaccharides is dominant, with less malic acid accumulated in the fruit at 10 ∼20 DAP. In addition, wax gourd fruit organic acid continued to accumulate until maturity without reducing, which is different from the accumulation pattern of other crops, such as watermelon, melon (Burger et al., 2003; Muhammad Jawad et al., 2020).

The processes involved in the metabolism and accumulation of organic acid in fruit are under genetic control. Many studies have helped decipher some of the mechanisms that control acidity. MDH catalyzes the reversible conversion of malate into OAA, the most likely direction being the synthesis of malic acid (Sweetman et al., 2009; Etienne et al., 2013). Based on our research, two *MDH* genes (*Bhi12G001426, Bhi01G000427*) critical for regulating malate accumulation, especially *MDH* (*Bhi12G001426*), which have more expression abundance among all homologous genes and were highly positive correlated with malic acid content in different wax gourd germplasms. In apple, the overexpression of *MdMDH* gene (GenBank Accession No. DQ221207), the homolog of wax gourd Bhi12G001426, contributed to malate accumulation in the apple callus, which supporting the involvement of *MdMDH* directly in malate synthesis (Yao et al., 2011; Yu et al., 2021). Phosphoenolpyruvate carboxylase (PEPC) was also indicated as the key enzyme in fruit malic acid synthesis (Etienne et al., 2013). In our study, the expression of two *PEPC*-related genes (*Bhi12G000721, Bhi09G002867*) was positively correlated with the malic acid content of different germplasm resources. Homeostasis and accumulation of organic acids depend on synthesis and degradation. Malic enzyme (ME) is considered an important enzyme involved in malic acid degradation, of which, *ME* gene (*Bhi01G002616*) with the highest expressed abundance is highly negatively correlated with malate content among diverse germplasm resources. It was concluded that *MDH* (*Bhi12G001426*), two *PEPC*-related genes (*Bhi12G000721, Bhi09G002867*) and *ME* (*Bhi01G002616*) may participate in the regulation of malic acid and its accumulation.

### One argininosuccinate synthases as catabolism pathway gene dominates the citrulline accumulation

Citrulline is widely found in cucurbit crops (Fish and Bruton, 2010; Hartman et al., 2019; Joshi et al., 2019). Based on the comparative analysis of citrulline in seven cucurbit crops (watermelon, melon, cucumber, bitter gourd, pumpkin, loofah, and wax gourd), it was found that the content of citrulline in wax gourd is rich, ranking in the forefront of cucurbitaceae, second only to watermelon (unpublished data). Citrulline in wax gourd accumulates gradually with the fruit developmental period, which is consistent with the findings in watermelon (Guo et al., 2013; Joshi et al., 2019). According to the RNAseq profiles of Guo et al. (2013), the expression of citrulline synthesis gene *OTC* remained steady throughout the fruit development, however, genes involved in citrulline degradation, two *ASS* genes were highly downregulated during watermelon flesh development. The highly negative correlation between catabolic genes and citrulline concentration suggests that citrulline accumulation depended on decreased activities of citrulline degradation rather than increased synthesis (Joshi and Fernie, 2017). In our study, the expression of one *ASS*-related gene (*Bhi02M000401*) was negatively correlated with citrulline content of different germplasm resources. In addition, it had the highest expression abundance among *ASS* homologous genes. Therefore, it is inferred as the most core gene to regulate the accumulation of citrulline.

### Transcription factors were identified from coexpression network with key enzyme genes related to taste and nutrition metabolites

Weighted gene co-expression network analysis is useful for identifying networks of co-expressed genes. By correlating the patterns of transcript accumulation and metabolic enzyme genes associated with the taste and nutrition metabolites, we identified 80, 24 TFs in the “yellow,” “brown or purple” modules, respectively with correlation coefficient > 0.80, among which, TFs (Dof, NAC) in the “yellow” module with correlation coefficients greater than 0.95 have been previously reported to strongly influence sugar accumulation in pitayas and watermelon (Wang et al., 2021; Mou et al., 2022). And TFs (MYB, ERF, bHLH, NAC) in the “brown or purple” modules with correlation coefficients greater than 0.80 were shown previously involved in malic or citric acid accumulation (Li et al., 2016, 2017; Hu et al., 2017; Yu et al., 2021). To further characterize the transcription factors that putatively regulate taste and nutrition metabolites, the potential binding affinity for the promoters of metabolic enzyme genes need to be further validated.

## Conclusion

This study clarified the taste and nutritional composition of wax gourd, as well as the accumulation patterns of these substances during fruit development. Furthermore, based on the time-course transcriptome analysis, the expression correlation verification analysis among twenty-four germplasms with different metabolites contents, and expression abundance of homologous genes, finally dominant genes regulating wax gourd monosaccharides, organic acids, and citrulline were identified. Understanding the core genes that influence the concentration of these substances deciding the taste and nutrition in fruit cells is thus of primary importance for fruit quality improvement.

## Data availability statement

The original contributions presented in this study are publicly available. This data can be found here: NCBI, PRJNA857066.

## Author contributions

SX, YZ, and DX conceived and designed the experiments. SX and XW performed the experiments. SX, XW, SL, YZ, and DX analyzed the data. SX wrote the manuscript. XW, SL, YZ, and DX reviewed and revised the manuscript. All authors have read and approved the final version of the manuscript.

## Footnotes

1
http://cucurbitgenomics.org/v2/ftp/genome/WaxGourd/

## Supplementary material

Supplementary Figure 1 Principle component analysis (PCA) of time-course transcriptome data.

Supplementary Figure 2 Validation of RNA-Seq results by qRT-PCR. The relative expression levels of six genes related with sugar, organic acid, citrulline metabolic pathways in five development stages of B variety by RNA-Seq using FPKM method and by qRT-PCR using the 2^–△△Ct^ method.

Supplementary Figure 3 The expression of other genes related to sugar were compared among numerous germplasm resources with different sugar content.

Supplementary Figure 4 The expression of other genes related to malic acid were compared among numerous germplasm resources with different malic acid content.

Supplementary Figure 5 The expression of other genes related to citrulline were compared among numerous germplasm resources with different citrulline content.

Supplementary Figure 6 Dendrogram showing co-expression modules (clusters) identified by weighted correlation network analysis (WGCNA) across fruit developmental stages. The major tree branches constitute 21 modules labeled with different colors.

## References

[B1] Aslam A. Zhao S. Azam M. Lu X. He N. Li B. (2020). Comparative analysis of primary metabolites and transcriptome changes between ungrafted and pumpkin-grafted watermelon during fruit development. *PeerJ* 8:e8259. 10.7717/peerj.8259 31934503 PMC6951286

[B2] Burger Y. Sa’ar U. Distelfeld A. Katzir N. Yeselson Y. Shen S. (2003). Development of Sweet Melon (*Cucumis melo*) Genotypes Combining High Sucrose and Organic Acid Content. *J. Am. Soc. Hortic. Sci.* 128 537–540. 10.21273/jashs.128.4.0537 35581909

[B3] Caffall K. H. Mohnen D. (2009). The structure, function, and biosynthesis of plant cell wall pectic polysaccharides. *Carbohydr. Res.* 344 1879–1900. 10.1016/j.carres.2009.05.021 19616198

[B4] Carmi N. Zhang G. Petreikov M. Gao Z. Eyal Y. Granot D. (2003). Cloning and functional expression of alkaline α−galactosidase from melon fruit: Similarity to plant SIP proteins uncovers a novel family of plant glycosyl hydrolases. *Plant J.* 33 97–106. 12943544 10.1046/j.1365-313x.2003.01609.x

[B5] Chrost B. Schmitz K. (1997). Changes in soluble sugar and activity of α-galactosidases and acid invertase during muskmelon (*Cucumis melo L.*) fruit development. *J. Plant Physiol.* 151 41–50. 10.1016/s0176-1617(97)80034-x

[B6] Corrigan V. K. Irving D. E. Potter J. F. (2000). Sugars and sweetness in buttercup squash. *Food Qual. Prefer*. 11 313–322. 10.1016/S0950-3293(99)00077-4

[B7] Dai N. Cohen S. Portnoy V. Tzuri G. Harel-Beja R. Pompan-Lotan M. (2011). Metabolism of soluble sugars in developing melon fruit: A global transcriptional view of the metabolic transition to sucrose accumulation. *Plant Mol. Biol.* 76 1–18. 10.1007/s11103-011-9757-1 21387125

[B8] Dhillon N. P. S. Sanguansil S. Singh S. P. Masud M. A. T. Kumar P. Bharathi L. K. (2016). “Gourds: bitter, bottle, wax, snake, sponge and ridge,” in *Genetics and genomics of the cucurbitaceae*, eds Grumet R. Katzir N. Garcia-Mas J. (New York, NY: Springer Intl Pub AG), 155–172. 10.1007/7397_2016_24

[B9] Doshi G. M. Nalawade V. V. Mukadam A. S. Chaskar P. K. Zine S. P. Somani R. R. (2015). Structural elucidation of chemical constituents from *Benincasa hispida* seeds and *Carissa congesta* roots by gas chromatography: Mass spectroscopy. *Pharmacogn. Res*. 7 282–93. 10.4103/0974-8490.157179 26130941 PMC4471656

[B10] Doty T. (1976). Fructose sweetness: A new dimension. *Cereal Foods World* 21 62–63.

[B11] Etienne A. Genard M. Lobit P. Mbeguie A. M. D. Bugaud C. (2013). What controls fleshy fruit acidity? A review of malate and citrate accumulation in fruit cells. *J. Exp. Bot.* 64 1451–1469. 10.1093/jxb/ert035 23408829

[B12] Fish W. Bruton B. (2010). “Quantification of L-citrulline and other physiologic amino acids in watermelon and selected cucurbits,” in *Proceedings of the Cucurbitaceae*, (Charleston), 152–154. 10.1179/1758897911Y.0000000001

[B13] Flores F. B. Martínez-Madrid M. C. Sánchez-Hidalgo F. J. Romojaro F. (2001). Differential rind and pulp ripening of transgenic antisenseACC oxidase melon. *Plant Physiol. Biochem.* 39 37–43. 10.1016/S0981-9428(00)01210-9

[B14] Gao L. Zhao S. Lu X. He N. Zhu H. Dou J. (2018). Comparative transcriptome analysis reveals key genes potentially related to soluble sugar and organic acid accumulation in watermelon. *PLoS One* 13:e0190096. 10.1371/journal.pone.0190096 29324867 PMC5764247

[B15] Gao Z. Schaffer A. A. (1999). A novel alkaline α-galactosidase from melon fruit with a substrate preference for raffinose. *Plant Physiol.* 119 979–988. 10.1104/pp.119.3.979 10069835 PMC32111

[B16] Guo S. Zhang J. Sun H. Salse J. Lucas W. J. Zhang H. (2013). The draft genome of watermelon (*Citrullus lanatus*) and resequencing of 20 diverse accessions. *Nat. Genet.* 45 51–58. 10.1038/ng.2470 23179023

[B17] Hartman J. Wehner T. Ma G. Perkins-Veazie P. (2019). Citrulline and Arginine Content of Taxa of Cucurbitaceae. *Horticulturae* 5:22. 10.3390/horticulturae5010022

[B18] Hu D. G. Li Y. Y. Zhang Q. Y. Li M. Sun C. H. Yu J. Q. (2017). The R2R3-MYB transcription factor MdMYB73 is involved in malate accumulation and vacuolar acidification in apple. *Plant J*. 91 443–454. 10.1111/tpj.13579 28423209

[B19] Hu L. P. Meng F. Z. Wang S. H. Sui X. L. Li W. Wei Y. X. (2009). Changes in carbohydrate levels and their metabolic enzymes in leaves, phloem sap and mesocarp during cucumber (*Cucumis sativus L.*) fruit development. *Sci. Hortic*. 121 131–137. 10.1016/j.scienta.2009.01.023

[B20] Huang H. Y. Huang J. J. Tso T. K. Tsai Y. C. Chang C. K. (2004). Antioxidant and angiotension-converting enzyme inhibition capacities of various parts of *Benincasa hispida* (wax gourd). *Nahrung* 48 230–233. 10.1002/food.200300428 15285118

[B21] Jayaprakasha G. K. Chidambara Murthy K. N. Patil B. S. (2011). Rapid HPLC-UV method for quantification of l-citrulline in watermelon and its potential role on smooth muscle relaxation markers. *Food Chem.* 127 240–248. 10.1016/j.foodchem.2010.12.098

[B22] Joshi V. Fernie A. R. (2017). Citrulline metabolism in plants. *Amino Acids* 49 1543–1559. 10.1007/s00726-017-2468-4 28741223

[B23] Joshi V. Joshi M. Silwal D. Noonan K. Rodriguez S. Penalosa A. (2019). Systematized biosynthesis and catabolism regulate citrulline accumulation in watermelon. *Phytochemistry* 162 129–140. 10.1016/j.phytochem.2019.03.003 30884257

[B24] Kim D. Langmead B. Salzberg S. L. (2015). HISAT: A fast spliced aligner with low memory requirements. *Nat. Methods* 12 357–360. 10.1038/nmeth.3317 25751142 PMC4655817

[B25] Kumar A. Vimalavathini R. (2004). Possible anorectic effect of methanol extract of *Benincasa hispida* (Thunb). Cogn, fruit. *Indian J. Pharmacol.* 36 348–350.

[B26] Leach D. N. Sarafis V. Spooner-Hart R. Wyllie S. G. (1989). Chemical and Biological Parameters of Some Cultivars of *Cucumis Melo*. *Acta Hortic.* 247, 353–358. 10.17660/ActaHortic.1989.247.68

[B27] Li S. J. Yin X. R. Wang W. L. Liu X. F. Zhang B. Chen K. S. (2017). Citrus CitNAC62 cooperates with CitWRKY1 to participate in citric acid degradation via up-regulation of *CitAco3*. *J. Exp. Bot.* 68 3419–3426. 28633340 10.1093/jxb/erx187 PMC5853897

[B28] Li S. J. Yin X. R. Xie X. L. Allan A. C. Ge H. Shen S. L. (2016). The Citrus transcription factor, CitERF13, regulates citric acid accumulation via a protein-protein interaction with the vacuolar proton pump, CitVHA-c4. *Sci. Rep*. 6:20151. 10.1038/srep20151 26837571 PMC4738278

[B29] Li X. Du J. Guo J. Wang H. Ma S. Lu J. (2018). The functions of cucumber sucrose phosphate synthases 4 (*CsSPS4*) in carbon metabolism and transport in sucrose- and stachyose-transporting plants. *J. Plant Physiol.* 228 150–157. 10.1016/j.jplph.2018.05.013 29913429

[B30] Li Y. Liu H. Yao X. Wang J. Feng S. Sun L. (2021). Hexose transporter CsSWEET7a in cucumber mediates phloem unloading in companion cells for fruit development. *Plant Physiol.* 186 640–654. 10.1093/plphys/kiab046 33604597 PMC8154047

[B31] Liu H. Liu X. Zhao Y. Nie J. Yao X. Lv L. (2022). Alkaline alpha-galactosidase 2 (*CsAGA2*) plays a pivotal role in mediating source-sink communication in cucumber. *Plant Physiol.* 189 1501–1518. 10.1093/plphys/kiac152 35357489 PMC9237694

[B32] Mou Z. L. Zeng R. X. Chen N. H. Liu Z. L. Zeng Z. X. Qin Y. H. (2022). The association of HpDof1. 7 and HpDof5. 4 with soluble sugar accumulation in pitaya fruit by transcriptionally activating sugar metabolic genes. *Food Qual. Saf*. 6:fyac042.

[B33] Muhammad Jawad U. Gao L. Gebremeskel H. Safdar L. B. Yuan P. Zhao S. (2020). Expression pattern of sugars and organic acids regulatory genes during watermelon fruit development. *Sci. Hortic.* 265:109102. 10.1016/j.scienta.2019.109102

[B34] Nakashima M. Shigekuni Y. Obi T. Shiraishi M. Miyamoto A. Yamasaki H. (2011). Nitric oxide-dependent hypotensive effects of wax gourd juice. *J. Ethnopharmacol.* 138 404–407. 10.1016/j.jep.2011.09.027 21963558

[B35] Nawirska-Olszanska A. Biesiada A. Sokol-Letowska A. Kucharska A. Z. (2014). Characteristics of organic acids in the fruit of different pumpkin species. *Food Chem.* 148 415–419. 10.1016/j.foodchem.2013.10.080 24262577

[B36] Obando-Ulloa J. M. Eduardo I. Monforte A. J. Fernández-Trujillo J. P. (2009). Identification of QTLs related to sugar and organic acid composition in melon using near-isogenic lines. *Sci. Hortic.* 121 425–433. 10.1016/j.scienta.2009.02.023

[B37] Palamthodi S. Lele S. S. (2014). Nutraceutical applications of gourd family vegetables: *Benincasa hispida, Lagenaria siceraria* and *Momordica charantia*. *Biomed. Prev. Nutr*. 4 15–21. 10.1016/j.bionut.2013.03.004

[B38] Pangborn R. M. (1963). Relative Taste Intensities of Selected Sugars and Organic Acids a. *J. Food Sci.* 28 726–733. 10.1111/j.1365-2621.1963.tb01680.x

[B39] Pharr D. M. Sox H. N. (1984). Changes in carbohydrate and enzyme levels during the sink to source transition of leaves of *Cucumis sativus* L., a stachyose translocator. *Plant Sci. lett*. 35 187–193. 10.1016/0304-4211(84)90227-X

[B40] Ren Y. Li M. Guo S. Sun H. Zhao J. Zhang J. (2021). Evolutionary gain of oligosaccharide hydrolysis and sugar transport enhanced carbohydrate partitioning in sweet watermelon fruits. *Plant Cell* 33 1554–1573. 10.1093/plcell/koab055 33570606 PMC8254481

[B41] Rennie E. A. Turgeon R. (2009). A comprehensive picture of phloem loading strategies. *Proc. Natl. Acad. Sci. U.S.A.* 106 14162–14167. 10.1073/pnas.0902279106 19666555 PMC2729037

[B42] Scherer R. Rybka A. C. P. Ballus C. A. Meinhart A. D. Filho J. T. Godoy H. T. (2012). Validation of a HPLC method for simultaneous determination of main organic acids in fruits and juices. *Food Chem.* 135 150–154. 10.1016/j.foodchem.2012.03.111

[B43] Sun X. Baldwin E. Plotto A. Cameron R. Manthey J. Dorado C. (2018). The effect of cultivar and processing method on the stability, flavor, and nutritional properties of winter melon juice. *Lwt* 97 223–230. 10.1016/j.lwt.2018.06.059

[B44] Sweetman C. Deluc L. G. Cramer G. R. Ford C. M. Soole K. L. (2009). Regulation of malate metabolism in grape berry and other developing fruits. *Phytochemistry* 70 1329–1344. 10.1016/j.phytochem.2009.08.006 19762054

[B45] Tang M. Bie Z.-L. Wu M.-Z. Yi H.-P. Feng J.-X. (2010). Changes in organic acids and acid metabolism enzymes in melon fruit during development. *Sci. Hortic.* 123 360–365. 10.1016/j.scienta.2009.11.001

[B46] Umer M. J. Bin Safdar L. Gebremeskel H. Zhao S. Yuan P. Zhu H. (2020). Identification of key gene networks controlling organic acid and sugar metabolism during watermelon fruit development by integrating metabolic phenotypes and gene expression profiles. *Hortic. Res*. 7:193. 10.1038/s41438-020-00416-8 33328462 PMC7705761

[B47] Wang J. Wang Y. Zhang J. Ren Y. Li M. Tian S. (2021). The NAC transcription factor *ClNAC68* positively regulates sugar content and seed development in watermelon by repressing *ClINV* and *ClGH3.6*. *Hortic. Res*. 8:214. 10.1038/s41438-021-00649-1 34593776 PMC8484586

[B48] Wang Y. Wyllie S. G. Leach D. N. (1996). Chemical Changes during the Development and Ripening of the Fruit of *Cucumis melo* (Cv. Makdimon). *J. Agric. Food Chem.* 44 210–216. 10.1021/jf9503568

[B49] Winter G. Todd C. D. Trovato M. Forlani G. Funck D. (2015). Physiological implications of arginine metabolism in plants. *Front. Plant Sci*. 6:534. 10.3389/fpls.2015.00534 26284079 PMC4520006

[B50] Xie D. Xu Y. Wang J. Liu W. Zhou Q. Luo S. (2019). The wax gourd genomes offer insights into the genetic diversity and ancestral cucurbit karyotype. *Nat. Commun.* 10:5158. 10.1038/s41467-019-13185-3 31727887 PMC6856369

[B51] Yamaguchi S. Yoshikawa T. Ikeda S. Ninomiya T. (1970). Studies on the taste of some sweet substances: Part I. Measurement of the relative sweetness part II. Interrelationships among them. *Agric. Biol. Chem.* 34 181–197. 10.1080/00021369.1970.10859599

[B52] Yao Y. X. Li M. Zhai H. You C. X. Hao Y. J. (2011). Isolation and characterization of an apple cytosolic malate dehydrogenase gene reveal its function in malate synthesis. *J. Plant Physiol.* 168 474–480. 10.1016/j.jplph.2010.08.008 20934777

[B53] Yu J. Q. Gu K. D. Sun C. H. Zhang Q. Y. Wang J. H. Ma F. F. (2021). The apple bHLH transcription factor *MdbHLH3* functions in determining the fruit carbohydrates and malate. *Plant Biotechnol. J.* 19 285–299. 10.1111/pbi.13461 32757335 PMC7868978

[B54] Zhang C. Yu X. Ayre B.G. Turgeon R. (2012). The origin and composition of cucurbit “Phloem” exudate. *Plant Physiol*. 158, 1873–1882. 10.1104/pp.112.194431 22331409 PMC3320192

[B55] Zhong Y. J. Zhou Y. Y. Li J. X. Yu T. Wu T. Q. Luo J. N. (2017). A high-density linkage map and QTL mapping of fruit-related traits in pumpkin (*Cucurbita moschata Duch.*). *Sci. Rep*. 7:12785. 10.1038/s41598-017-13216-3 28986571 PMC5630576

