# Supplementary figures and images for: A time-course transcriptome analysis of wax gourd fruit development reveals predominant genes regulating taste and nutrition
Source: Front Plant Sci. 2022 Sep 8;13:971274. doi: 10.3389/fpls.2022.971274 (PMC9493329; doi:10.3389/fpls.2022.971274)

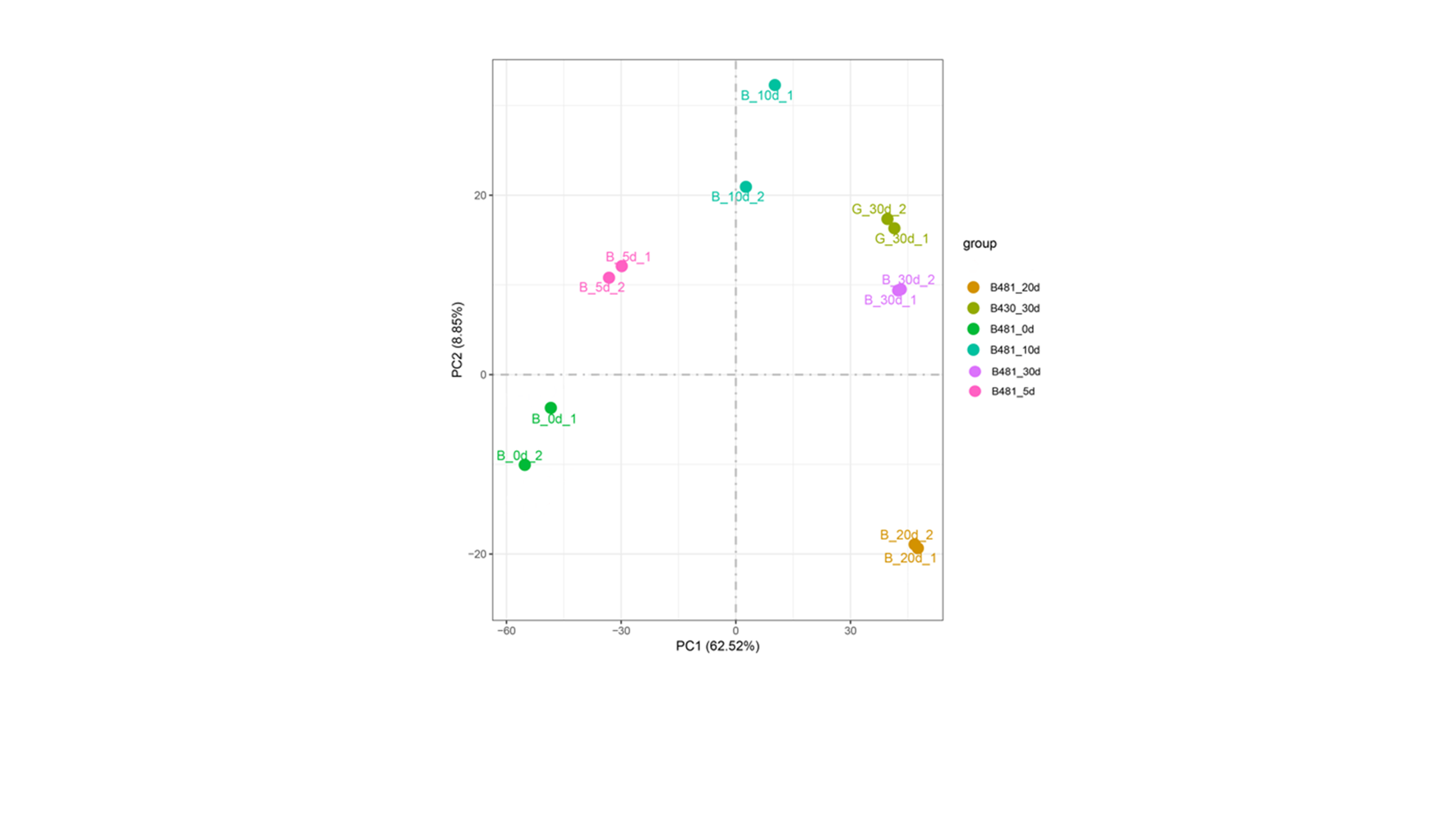

Supplement: Supplementary Figure 1 — Principle component analysis (PCA) of time-course transcriptome data. [file Image_1.TIF]

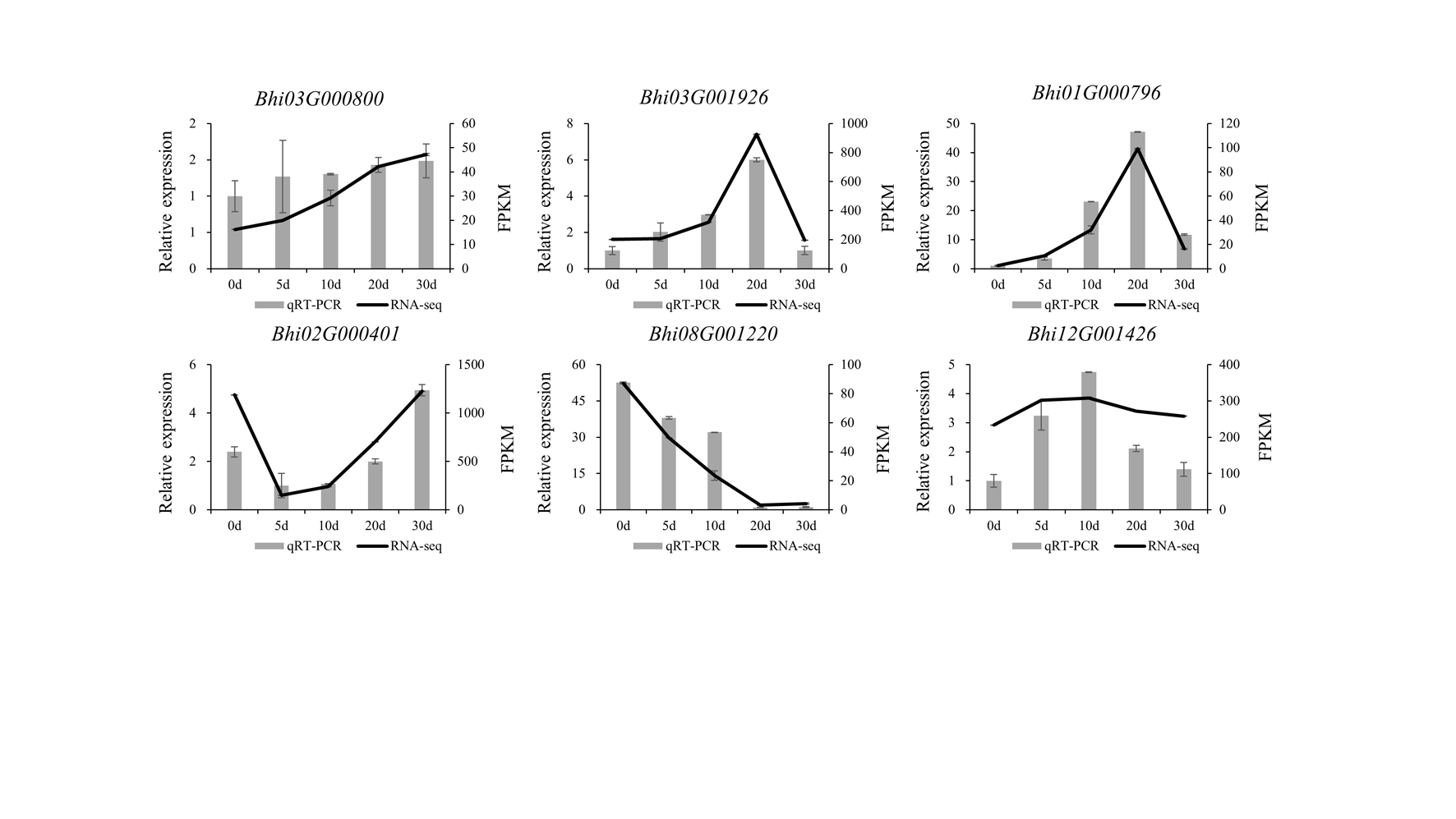

Supplement: Supplementary Figure 2 — Validation of RNA-Seq results by qRT-PCR. The relative expression levels of six genes related with sugar, organic acid, citrulline metabolic pathways in five development stages of B variety by RNA-Seq using FPKM method and by qRT-PCR using the 2–△△Ct method. [file Image_2.TIF]

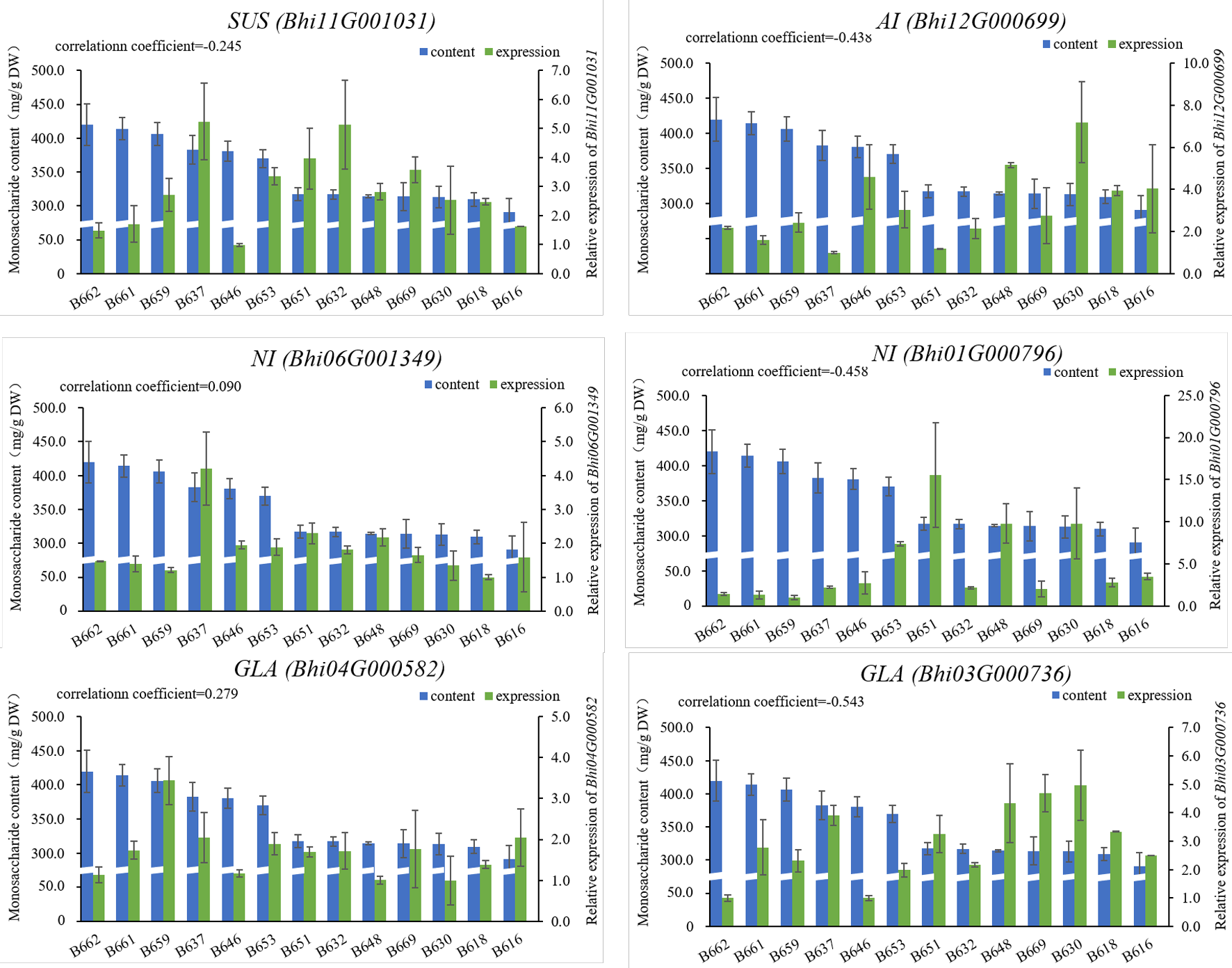

Supplement: Supplementary Figure 3 — The expression of other genes related to sugar were compared among numerous germplasm resources with different sugar content. [file Image_3.TIF]

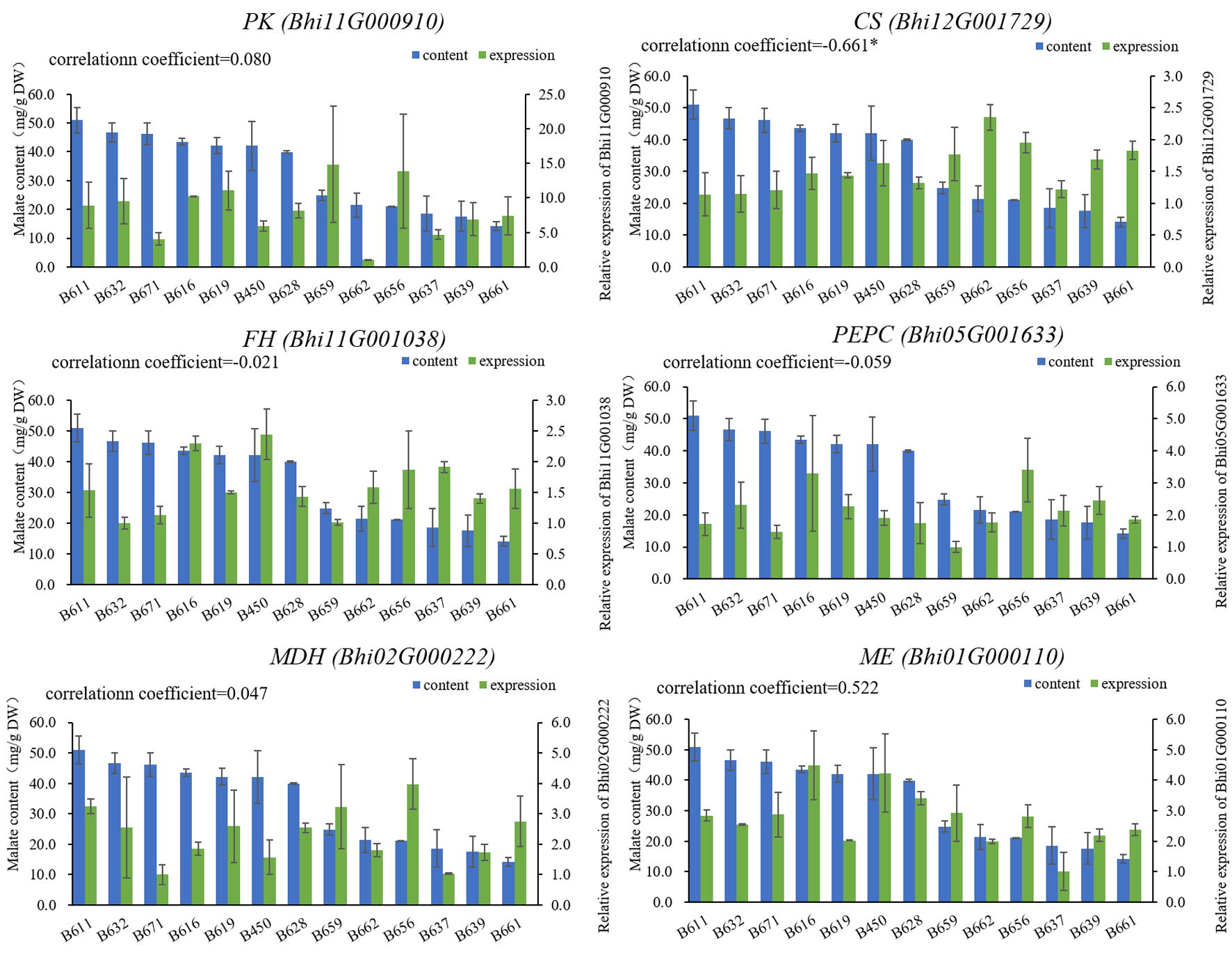

Supplement: Supplementary Figure 4 — The expression of other genes related to malic acid were compared among numerous germplasm resources with different malic acid content. [file Image_4.TIF]

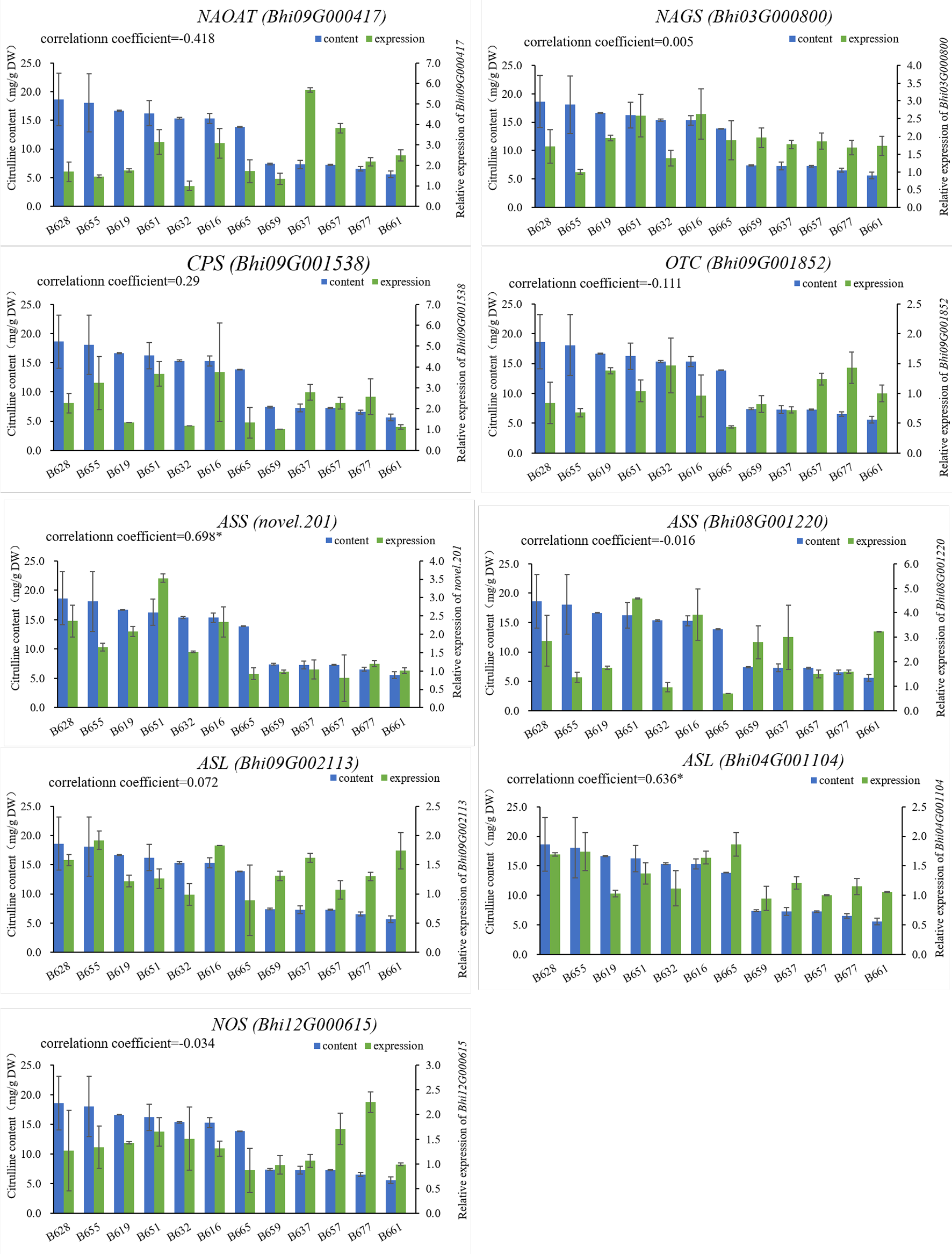

Supplement: Supplementary Figure 5 — The expression of other genes related to citrulline were compared among numerous germplasm resources with different citrulline content. [file Image_5.TIF]

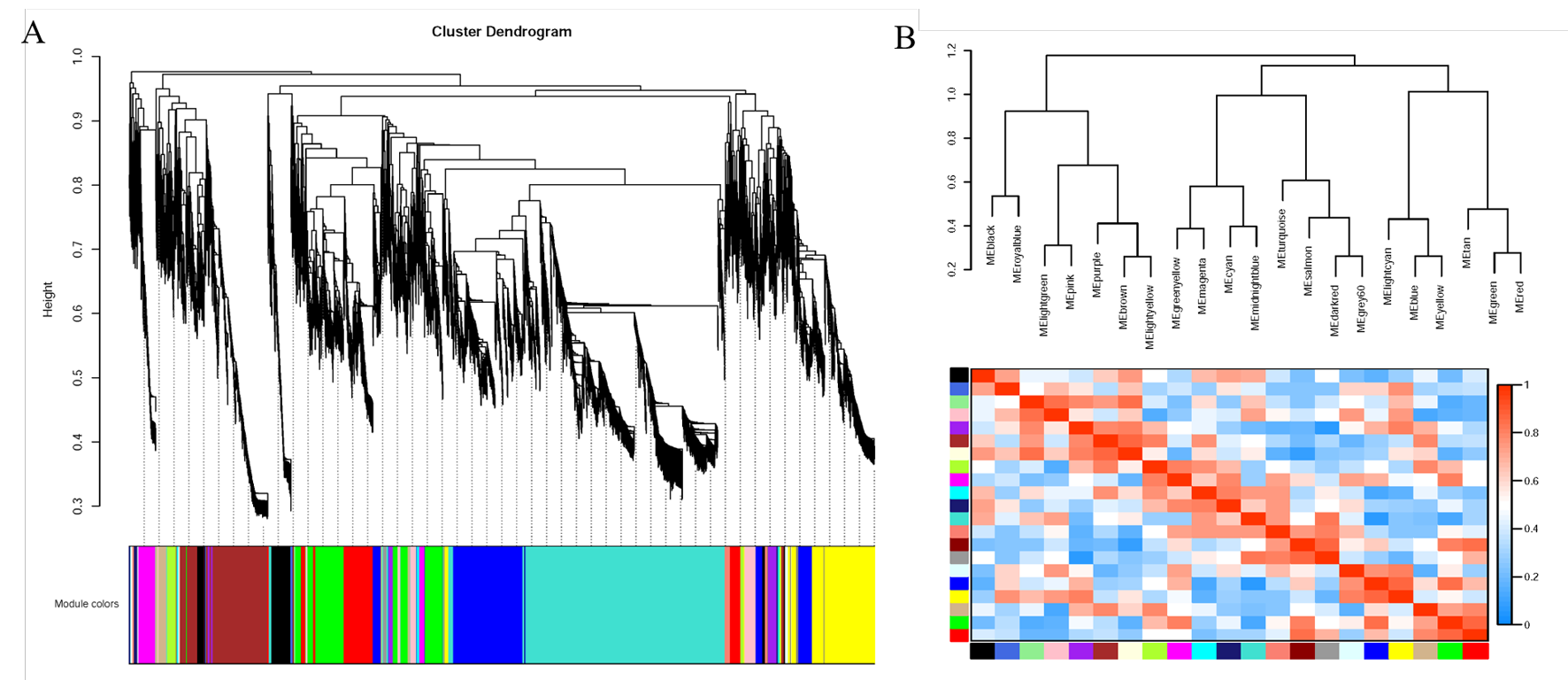

Supplement: Supplementary Figure 6 — Dendrogram showing co-expression modules (clusters) identified by weighted correlation network analysis (WGCNA) across fruit developmental stages. The major tree branches constitute 21 modules labeled with different colors. [file Image_6.TIF]
